# Baseline cerebral structural morphology predict freezing of gait in early drug-naïve Parkinson’s disease

**DOI:** 10.1038/s41531-022-00442-4

**Published:** 2022-12-29

**Authors:** Yuting Li, Xiaofei Huang, Xiuhang Ruan, Dingna Duan, Yihe Zhang, Shaode Yu, Amei Chen, Zhaoxiu Wang, Yujian Zou, Mingrui Xia, Xinhua Wei

**Affiliations:** 1 Department of Radiology, the Second Affiliated Hospital, School of Medicine, South China University of Technology, Guangdong, China; 2 Affiliated Dongguan Hospital, Southern Medical University (Dongguan People’s Hospital), Guangdong, China; 3 State Key Laboratory of Cognitive Neuroscience and Learning, Beijing Normal University, Beijing, China; 4 School of Information and Communication Engineering, Communication University of China, Beijing, China

**Keywords:** Parkinson's disease, Movement disorders, Magnetic resonance imaging, Predictive markers

## Abstract

Freezing of gait (FOG) greatly impacts the daily life of patients with Parkinson’s disease (PD). However, predictors of FOG in early PD are limited. Moreover, recent neuroimaging evidence of cerebral morphological alterations in PD is heterogeneous. We aimed to develop a model that could predict the occurrence of FOG using machine learning, collaborating with clinical, laboratory, and cerebral structural imaging information of early drug-naïve PD and investigate alterations in cerebral morphology in early PD. Data from 73 healthy controls (HCs) and 158 early drug-naïve PD patients at baseline were obtained from the Parkinson’s Progression Markers Initiative cohort. The CIVET pipeline was used to generate structural morphological features with T1-weighted imaging (T1WI). Five machine learning algorithms were calculated to assess the predictive performance of future FOG in early PD during a 5-year follow-up period. We found that models trained with structural morphological features showed fair to good performance (accuracy range, 0.67–0.73). Performance improved when clinical and laboratory data was added (accuracy range, 0.71–0.78). For machine learning algorithms, elastic net-support vector machine models (accuracy range, 0.69–0.78) performed the best. The main features used to predict FOG based on elastic net-support vector machine models were the structural morphological features that were mainly distributed in the left cerebrum. Moreover, the bilateral olfactory cortex (OLF) showed a significantly higher surface area in PD patients than in HCs. Overall, we found that T1WI morphometric markers helped predict future FOG occurrence in patients with early drug-naïve PD at the individual level. The OLF exhibits predominantly cortical expansion in early PD.

## Introduction

It has attracted increasing attention that in advanced disease stages, most patients with Parkinson’s disease (PD) suffer from a crippling gait disorder: freezing of gait (FOG)^1^. This gait disturbance greatly interferes with the daily life of patients with PD. More importantly, FOG is now considered to be one of the main risk factors for falls and contributes to increased emotional disorders in PD patients^1–3^. Therefore, it could significantly weaken the movement ability and diminish the quality of life in PD patients. Some researchers have found that compared with PD patients without FOG and healthy people, the gray matter (GM) of several brain regions related to motor, executive attention, and cognition have different degrees of atrophy in PD patients with FOG^4–6^. At present, the treatment of FOG is still extremely challenging, and there is no unified and effective treatment in the clinic, let alone a cure^7^. Therefore, it is of great importance to predict FOG in the early stage of the disease for prevention and intervention in PD patients.

Recent studies have suggested that clinical assessments, laboratory tests, and brain imaging of early PD patients could predict the progression of dyskinesias^8^, as well as the occurrence of postural instability and gait difficulties (PIGD)^9^ and even FOG^10–12^ with generalized linear models or logistic regression models. However, these studies have only focused on a certain brain area or have a small sample. What’s more, combining clinical, laboratory, and imaging data to predict the occurrence of FOG using machine learning has not previously been undertaken in early drug-naïve PD patients.

Additionally, previous imaging studies have shown that cerebral structural morphology changes in PD are mainly located in brain regions related to dopamine transport pathways, such as the temporo-occipital lobe, and part of the frontoparietal lobe^13–15^. However, there is considerable heterogeneity in the current findings, while the localization and extent of PD-related cortical damage and/or white matter (WM) abnormalities still need to be further explored.

Here, we developed a model that could predict the occurrence of FOG at the individual level using machine learning with clinical assessments, laboratory tests and cerebral structural imaging information of early drug-naïve PD patients. As a secondary objective, we explored the morphological alterations of the cerebrum in early drug-naïve PD patients and their relationship with clinical and laboratory assessments.

## Results

### Demographic, clinical, and CSF characteristics of the participants

**Fig. 1:**
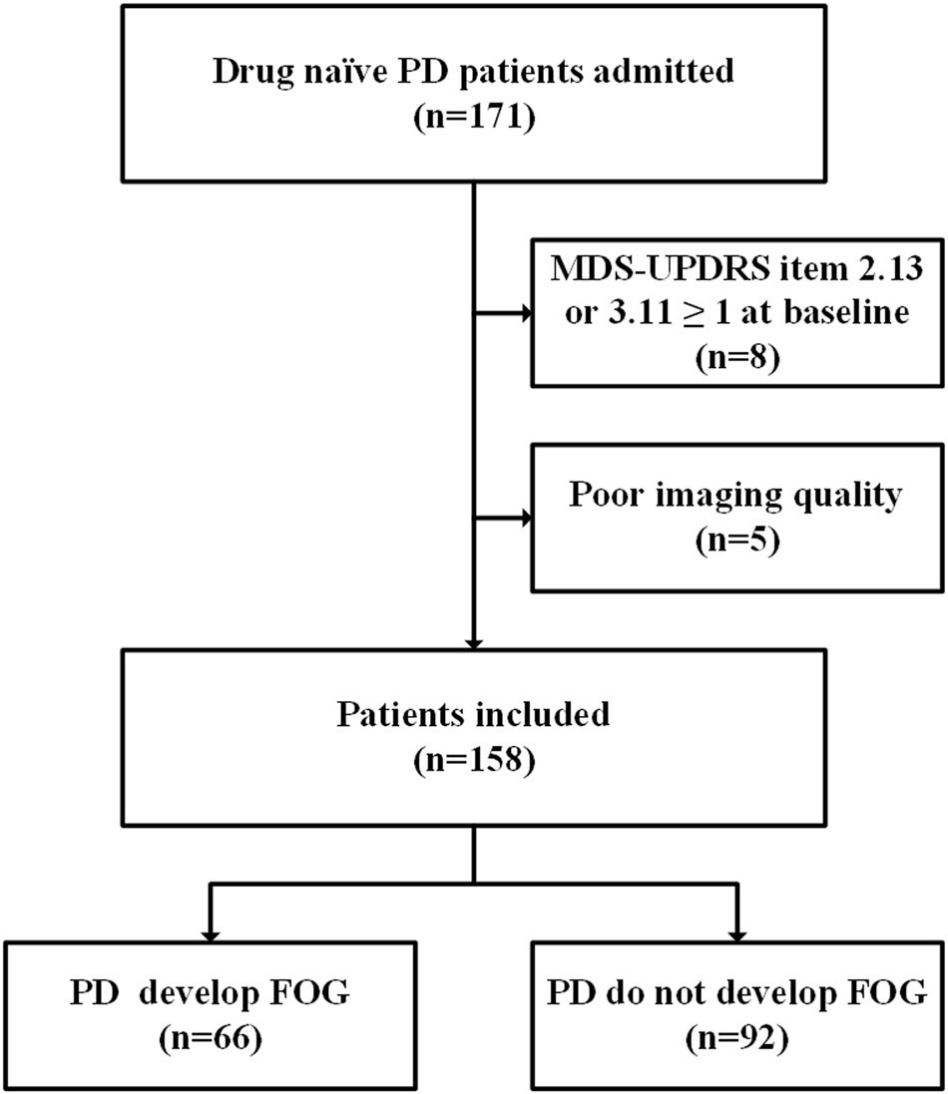
Flow chart illustrating patient selection.

A total of 73 HCs (65.8% males) and 158 participants with drug-naïve PD (63.3% males) were included in the analyses, and 66 (74.2% males) patients in the PD cohort developed FOG during the follow-up period (Fig. 1). The median time to the first occurrence of FOG was 29 (2–96) months from baseline. Table 1 summarizes the baseline demographics and clinical and laboratory characteristics of the study population. Briefly, the PD patients suffered from sleep disorder, olfactory dysfunction, anxiety, depression, and increasing p-tau/t-tau of CSF at baseline. Moreover, compared to the participants with PD who did not develop FOG, the PD patients who developed FOG had decreased olfactory function, symptoms of depression, a more severe disease degree, dysfunction of daily living and movement, postural instability, and gait difficulty at baseline. It seems that men are more prone to FOG than women. However, no statistically significant difference was noted between the two groups in laboratory assessments, including CSF, urate, and APOE4.

**Table 1:** Baseline demographics, clinical and laboratory characteristics of all participants.

	PD do not develop FOG (92)	PD develop FOG (66)	*P* value	PD (158)	HCs (73)	*P* value
Age	60.47 (9.969)	63.18 (8.709)	0.104	61.06 (9.746)	60.19 (10.629)	0.278
Sex (male/female)	51/41	49/17	0.016*	100/58	48/25	0.717
Education (yr)	15.34 (3.010)	15.41 (2.972)	0.999	15.37 (2.985)	15.60 (3.036)	0.691
Handed (R/L/Both)	81/6/5	60/3/3	0.836	141/9/8	59/7/7	0.215
MOCA	28.25 (1.860)	27.51 (1.816)	0.057	27.93 (1.863)	28.39 (1.204)	0.238
RBDSQ	3.56 (2.315)	4.57 (2.917)	0.173	4.04 (2.654)	2.83 (2.135)	<0.001*
UPSIT	22.95 (7.716)	20.06 (10.005)	0.018*	21.77 (8.802)	34.15 (4.240)	<0.001*
STAI	62.31 (17.540)	67.91 (19.313)	0.113	65.10 (18.868)	56.67 (13.030)	0.003*
GDS	1.81 (1.790)	2.51 (2.439)	0.035*	2.17 (2.197)	1.13 (2.155)	<0.001*
QUIP	0.19 (0.588)	0.11 (0.375)	0.846	0.15 (0.506)	0.17 (0.637)	0.767
H&Y	1.469 (0.534)	1.72 (0.452)	0.019*	1.56 (0.510)	–	−
UPDRS-I	4.31 (3.075)	5.11(3.038)	0.095	5.07 (3.594)	−	−
UPDRS-II	3.92 (3.098)	6.91 (4.267)	<0.001*	5.35 (3.790)	−	−
UPDRS-III	18.47 (7.634)	25.36 (10.311)	0.001*	20.77 (8.926)	−	−
Tremor	0.46 (0.277)	0.44 (0.335)	0.078	0.44 (0.300)	−	−
PIGD	0.16 (0.193)	0.31 (0.235)	<0.001*	0.21 (0.234)	−	−
SCOPA-AUT	7.34 (4.191)	9.96 (6.960)	0.065	8.79 (5.756)	−	−
Aβ1-42 (e^2^)	9.13 (3.061)	9.06 (3.153)	0.523	9.08 (3.084)	9.90 (4.426)	0.395
α-syn (e^3^)	1.59 (0.721)	1.49 (0.555)	0.259	15.40 (6.552)	15.28 (5.571)	0.817
t-tau (e^2^)	1.71 (0.531)	1.71 (0.443)	0.558	1.71 (0.496)	1.821(0.694)	0.736
p-tau	14.66 (5.045)	14.36 (4.453)	0.625	1.44 (0.480)	1.57 (0.670)	0.265
t-tau/ Aβ1-42 (e^−2^)	19.95 (7.320)	20.79 (9.411)	0.633	20.27 (8.213)	19.74 (8.898)	0.235
p-tau/ Aβ 1-42 (e^−3^)	17.00 (6.880)	17.50 (8.760)	0.970	17.18 (7.676)	17.18 (8.990)	0.355
p-tau/t-tau (e−^3^)	84.70 (5.570)	83.40 (6.500)	0.081	84.14 (5.969)	86.07 (8.757)	0.013*
urate (e^2^)	3.06 (0.764)	3.29 (0.792)	0.074	3.16 (0.779)	3.21 (0.757)	0.843
APOE4	0.38 (0.577)	0.36 (0.568)	0.801	0.37 (0.569)	0.31 (0.507)	0.469

*PD* Parkinson’s disease, *FOG* freezing of gait, *HCs* healthy controls, *yrs* years, *R* right, *L* left, *MOCA* Montreal Cognitive Assessment, *RBDSQ* Rapid Eye Movement Sleep Behavior Disorder Screening Questionnaire, *UPSIT* University of Pennsylvania Smell Identification Test, *STAI* State-trait Anxiety Inventory, *GDS* Geriatric Depression Scale, *QUIP* Questionnaire for Impulsive-Compulsive Disorders in Parkinson’s Disease, *H&Y* Hoehn and Yahr Scale, *UPDRS I–III* Unified Parkinson’s disease Rating Scale Part I–III, *PIGD* postural instability and gait difficulties, *SCOPA-AUT* Scales for Outcomes in Parkinson’s Disease—Autonomic, *α-syn* α-synuclein.

*P < 0.05.

### Different models predict incident FOG

**Fig. 2:**
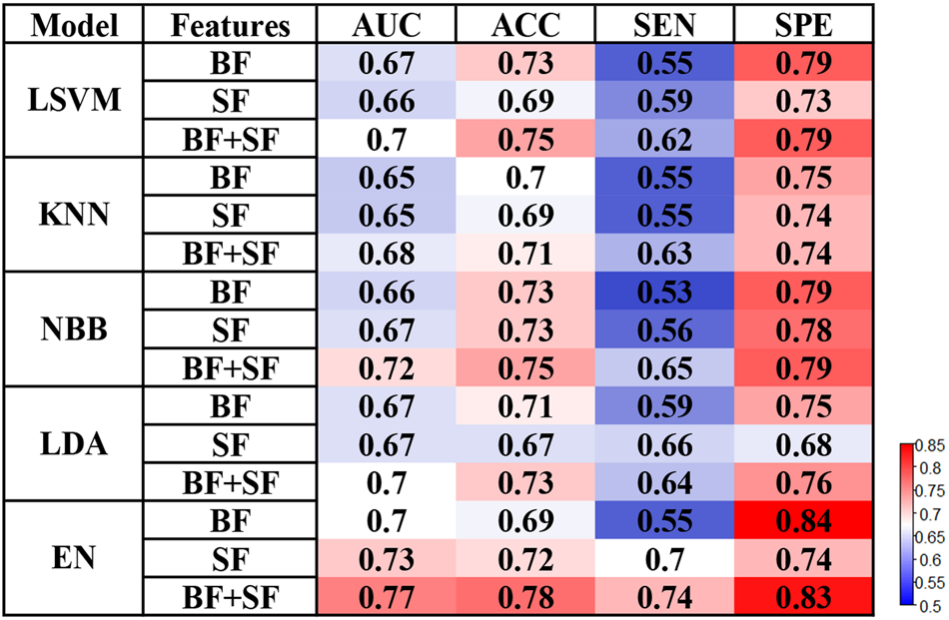
Freezing of gait prediction performances with various machine learning models and features.

Model performance is summarized in Fig. 2. The proposed predictors discriminated PD patients with FOG with fair prediction accuracy. The prediction models showed that combining clinical and laboratory evaluations with structural morphology features yielded better performance (AUC range, 0.67–0.77; ACC range, 0.71–0.78) than adding clinical and laboratory evaluations only (AUC range, 0.65–0.70; ACC range, 0.69–0.73) or structural morphology features only (AUC range, 0.65–0.73; ACC range, 0.67–0.73) in the prediction of the development of FOG. For machine learning algorithms, EN-SVM models (AUC range, 0.70–0.77; ACC range, 0.69–0.78) were generally better than the other four machine learning models (AUC range, 0.65–0.72; ACC range, 0.67–0.75). Additionally, the model performance of trials with the proportion of future FOG to non-FOG of 5:5 and 3:7 was better (Supplementary Table 2), but it should be noted that 4:6 is the original proportion of the samples in this study, and the model performance of it was only slightly lower than the former two.

### Weighting factors/feature selection

**Fig. 3:**
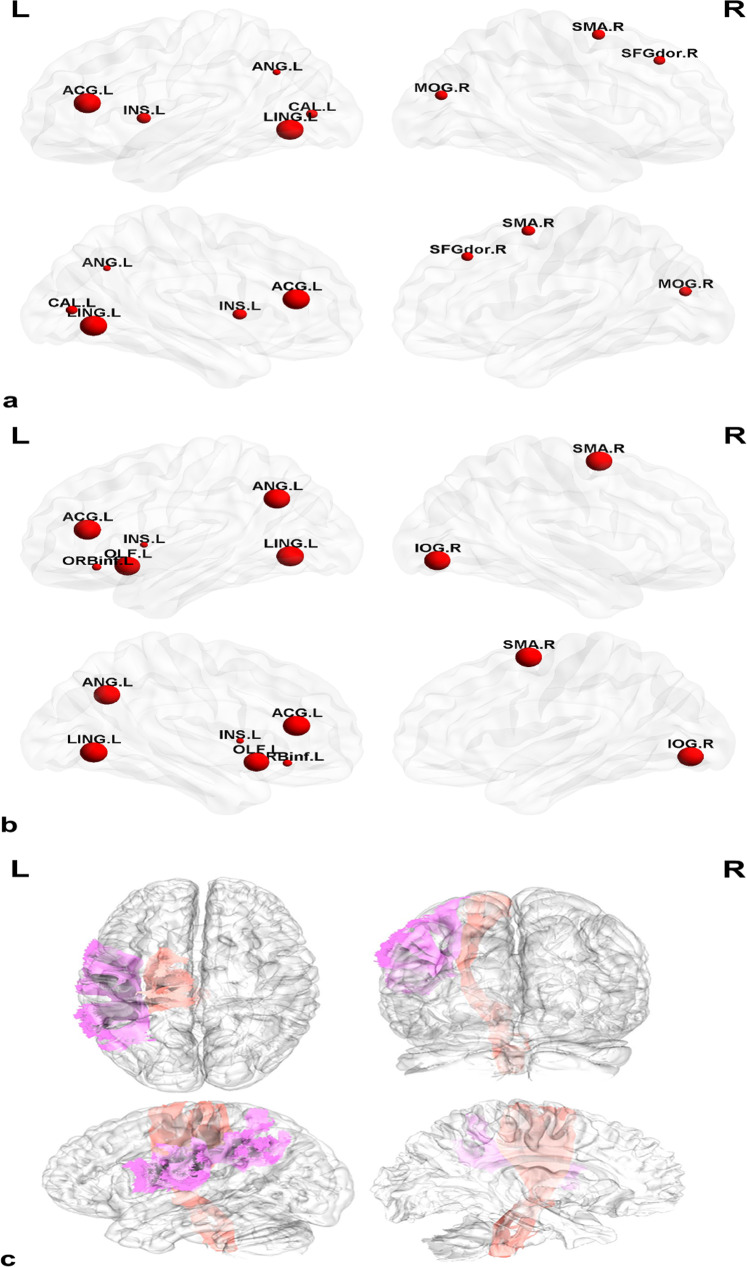
Structural weighting factors of the EN-SVM predictive model of FOG. **a** The main weighted features of the EN-SVM model using structural features. **b** The main weighted features of the EN-SVM model combing clinical, biological, and structural features. **c** The selected white matter of the EN-SVM model.

Weighting factors and selected features are summarized in Tables 2 and 3. Both prediction models based on EN-SVM using structural features with and without clinical and CSF features showed that the main features predicting FOG at baseline PD were the right supplementary motor area (SMA.R) and the left hemispheres (Fig. 3), mainly distributed in the lingual gyrus (LING.L), anterior cingulate and paracingulate gyri (ACG.L), angular gyrus (ANG.L), insula (INS), superior longitudinal fasciculus and corticospinal tract. Of note, the top 10 features of the EN-SVM model using structural features also included the right middle occipital gyrus (MOG.R), superior frontal gyrus, dorsolateral (SFGdor.R) and calcarine fissure, and the left surrounding cortex (CAL.L) (Fig. 3). Meanwhile, the top 10 features of the EN-SVM model combining clinical, laboratory and structural features also included the GM volumes of the right inferior occipital gyrus (IOG.R), the left olfactory cortex (OLF.L) and the left inferior frontal gyrus, orbital part (ORBinf.L) (Fig. 3). The UPDRS II and III as well as the PIGD were selected in the EN-SVM model using clinical and laboratory features.

**Table 2:** Top 10 features of the EN-SVM model using different features.

BF	Weight	SF	Structural features	Weight	All	Structural features	Weight
PIGD	0.0817111855246307	LING.L	Grey matter volume	1,612,037.999	LING.L	Grey matter volume	1,289,290.611
MDS-UPDRS-II	0.0166991619743191	ACG.L	Grey matter volume	1,350,838.573	ACG.L	Grey matter volume	1,092,129.818
MDS-UPDRS-III	0.00355655486563525	ANG.L	Grey matter volume	219,659.1359	SMA.R	Grey matter volume	332,300.2849
UPSIT	0.000232737314552627	Superior.longitudinal.fasciculus.(temporal.part).L	White matter volume	18,547.420	ANG.L	Grey matter volume	327,167.6149
		Corticospinal.tract.L	White matter volume	17,718.850	IOG.R	Grey matter volume	179,288.0583
		INS.L	Cortical mean curve	6.0294205	OLF.L	Grey matter volume	71,397.58969
		SMA.R	Cortical mean curve	4.7943908	Superior.longitudinal.fasciculus.(temporal.part).L	White matter volume	17,859.58454
		MOG.R	Cortical mean curve	3.476970156	Corticospinal.tract.L	White matter volume	16,401.73697
		SFGdor.R	Cortical mean curve	2.472278067	ORBinf.L	Cortical mean curve	4.47016179
		CAL.L	Cortical mean curve	2.328508489	INS.L	Cortical mean curve	3.42310076

*BF* both clinical and laboratory features, *SF* structural features, *R* right, *L* left, *PIGD* postural instability and gait difficulties, *UPDRS II–III* Unified Parkinson’s disease Rating Scale Part II–III, *UPSIT* University of Pennsylvania Smell Identification Test, *LING* lingual gyrus, *ACG* anterior cingulate and paracingulate gyri, *ANG* angular gyrus, *INS* insula, *SMA* supplementary motor area, *MOG* middle occipital gyrus, *SFGdor* superior frontal gyrus, dorsolateral, *CAL* calcarine fissure and the left surrounding cortex, *IOG* inferior occipital gyrus, *OLF* olfactory cortex, *ORBinf* inferior frontal gyrus, orbital part.

**Table 3:** The results of feature selection based on generalized Fisher score using different features.

Features	Mean	SD
*BF*		
MDS-UPDRS	1.70	0.84
MDS-UPDRS-I	2.63	1.35
PIGD	3.03	1.35
*SF*		
INS.L (cortical mean curvature)	2.73	2.38
MOG.R (cortical mean curvature)	12.17	17.68
*All*		
MDS-UPDRS	3.37	3.19
INS.L (cortical mean curvature)	3.87	3.12
MOG.R (cortical mean curvature)	10.50	13.36
PIGD	11.00	12.84
MDS-UPDRS-I	11.33	10.84

*BF* both clinical and laboratory features, *SF* structural features, *R* right, *L* left, *PIGD* postural instability and gait difficulties, *UPDRS I* unified Parkinson’s disease Rating Scale Part I, INS insula, *MOG* middle occipital gyrus.

In the other four machine-learning models, the total scores of UPDRS, UPDRS-I, and the PIGD were selected among the clinical and laboratory features, where the surface mean curvature of the INS.L and the MOG.R-consistent with the features selected from the EN-SVN model, were selected among the structural features.

Thus, the aberrance of structural morphology of multiple brain regions could jointly predict the occurrence of FOG in patients with PD, according to the indices of contribution to the model. Moreover, incorporating the UPDRS, UPDRS-I, and PIGD assessments allowed the prediction model to achieve a better discriminative ability.

### Structural morphology measurements and their relationship with clinical and laboratory assessments

Only the bilateral OLF (OLF.L: *p* = 0.039; OLF.R: *p* = 0.042) showed a significantly higher surface area in PD patients than in HCs (Supplementary Fig. 1a, Supplementary Table 3). Notably, the MOCA was associated with the surface area of the left OLF (*p* = 0.021, *r* = 0.186, Bonferroni-corrected) but not with the right OLF (Supplementary Fig. 1b) in PD patients. We found no significant difference in cortical thickness, surface means curvature, GM, or WM volumes in patients with PD compared to HCs. In addition, we found that compared with future non-FOG, there are alterations in structural measurements at baseline in future FOG. (Supplementary Table 4). It should be noted that none of the structural morphology measurements could be corrected by FDR.

## Discussion

We found that the decreasing GM volumes and the increasing surface mean curve of some brain regions could help predict future conversion to FOG in early drug-naïve PD patients at an individual level using machine learning. Of note, combining the structural features and the assessments of the UPDRS, UPDRS-I and the PIGD could result in better performance. Additionally, the bilateral OLF showed a significantly higher surface area in PD patients than in HCs, but only the surface area of the left OLF was associated with cognitive function: the MOCA.

The proposed model that uses structural morphology measurements (T1WI) achieved good predictive accuracy of FOG at baseline (AUC range, 0.65–0.73; ACC range, 0.67–0.73). It performed better when adding clinical and laboratory evaluations (AUC range, 0.68–0.77; ACC range, 0.71–0.78). The models developed in this study using structural features with and without clinical and laboratory features performed better than the previous study using clinical characteristics only^16^. Another study combining the PIGD score, caudal DAT uptake, and Aβ 1-42 of CSF to predict FOG reported an AUC of 0.755^17^, which was similar to the AUC of our study. However, neither the weighting factors nor the feature selection of our models selected CSF markers. The reason may be that the models we developed were different. Kim et al. used Cox proportional-hazards regression analyses, while we used the elastic net and the generalized Fisher score (GFS) for feature selection without fixed risk factors. In addition, it also found that the presynaptic striatum dopaminergic innervation (where the HR of DAT uptake in the caudate nucleus is 0.551, and the HR of DAT uptake in the putamen is 0.441) could predict the development of FOG in patients with new PD^11^. The above studies have a certain potential for the prediction of FOG. However, it is worth mentioning that DAT imaging is a radiographic imaging technology, and the collection of CSF is an invasive operation. We also found that there is a regularity in the different models that the behavioral features seem to be more specific than sensitive, while the structural features seem to be more sensitive than specific and the combination of the two is certainly additive, which suggested that the combination of behavioral and structural features had the potential guiding ability for predicting the occurrence of FOG in patients with early drug-naïve PD. Regardless of the features selected, the current models were not pretty good (AUC < 0.8). It is worth noting that, predicting the occurrence of FOG in the early stages of PD is inherently a challenging clinical issue. Here, we used machine learning algorithms that are simple, easy to implement, and highly interpretable. Further studies could explore whether the conversion to FOG could be predicted at the genetic and molecular level in patients with early PD.

Past studies with different cohorts found certain clinical risk factors for FOG, such as old age at PD onset, male sex, longer disease duration, lower baseline cognitive function, severe baseline motor symptoms, and depression^11,16–18^. Here, we found that the assessments of UPDRS, UPDRS-I, and the PIGD could potentially help predict FOG in patients with early drug-naïve PD, which was partially consistent with the findings of a previous study^19^. The items of the UPDRS were strongly correlated with specific FOG questionnaires developed more recently, such as the Gait and Falls Questionnaire and Freezing of Gait Questionnaire^20,21^. Actually, consistent with previous studies^10,11,17,18^, PD patients with future FOG reported more gait-related discomfort at baseline patient interview, suggesting possibly increased vulnerability and a greater underlying disease-related burden may exist in early PD. Moreover, the UPDRS-I scale assesses non-motor symptoms in daily life in PD patients, including cognition, hallucinations, depression, anxiety, apathy, sleeping, and autonomic symptoms. The presence of non-motor symptoms may suggest a potential occurrence of FOG in PD. Hence, the increase in the UPDRS, the UPDRS-I, and motion-related scores in early PD might help predict the future occurrence of FOG.

We found that several disrupted brain regions that might help predict future FOG were mainly distributed in the occipital lobe, limbic systems, and part of the frontoparietal lobes. Anatomically, the visual center is primarily located in the occipital lobe, whereas the lingual gyrus belongs to the first-order visual center. In addition, the visual cortex in the occipital lobe has fiber connections to the parietal, temporal, and angular gyri. A previous study found that PD patients with visual hallucinations suffered from more severe disruption of the frontal cortex than PD without visual hallucinations^22^. A meta-analysis showed that the severity of hallucinations in schizophrenia was associated with reduced GM in parts of the temporal lobe and bilateral supramarginal and angular gyri^23^. Therefore, decreasing GM volume in the left lingual and angular gyrus might lead to visual disturbances and even visual hallucinations, which may be related to the dysfunction in posterior visual processing networks in such patients^24^. When patients with PD suffer from visual disturbances and/or hallucinations, or a specific deficit of the visuospatial function, they are more likely to fall, and be with worse cognitive and executive function, thus causing greater fear of falling, leading to the possible development of FOG^24–26^. Moreover, the development of FOG is impacted by dysthymic disorders dominated by the limbic system, including the cingulate gyrus and insula. The disruption of the fronto-striato-limbic network might underpin the link between dysthymic disorders and FOG in PD. It is proposed that movement disorders have aggravated the striato-limbic load and reduced top-down attentional control at rest, which might lead to FOG when further challenged by the parallel processing demands of walking^27^. In addition, previous studies have illustrated that pathological damage to the caudate nucleus and frontal lobe might also be involved in the impact of psychological alterations on FOG and explain why FOG can be observed in patients with frontal impairments^28^. Notably, our study also found that the WM volume of the left superior longitudinal fasciculus and corticospinal tract makes some contribution to the predictive models of the occurrence of FOG. Several studies have found WM damage in both the superior longitudinal fasciculus and the corticospinal tract in PD patients with FOG. It is believed that the abnormality of the above fibers leads to abnormal connections between brain regions, indicating that PD patients with FOG might be the result of poor structural and functional integration of motor and extramotor neural systems^6,29^.

The results presented here suggest that in the bilateral OLF, early drug-naïve PD patients exhibit a larger cortical surface area. Zeighami et al. also used the PPMI database to map the distribution of atrophy in PD and found that besides subcortical areas, the medial temporal lobe, and discrete cortical regions were impaired in PD^15^, which was partially consistent with our findings. One possible reason is the inconsistency in inclusion criteria: they included data from all types of machines, while we only included data acquired from Siemens’ machines; Another possible reason is the inconsistency in analysis methods: they combined deformation-based morphometry and independent component analysis, focusing more on network structure, while we used surface-based morphometry and voxel-based morphometry methods, focusing more on brain region structures. Some researchers believe that “compensated hyperplasia” appears in specific brain regions in early PD, which might be related to the compensatory neuroinflammatory response^30,31^. Astrocytes are activated by proinflammatory cytokines, resulting in cell hypertrophy, astrocyte proliferation, protrusion extension, and interlacing, which leads to increasing surface area and/or thickness of the cortex^32^. What’s more, the highest density of cholinergic markers is existed in the striatum of the brain, and there is a cholinergic pathway between the striatum and the posterior cortex, including the OLF^33^. Some molecular imaging studies found that compensatory cholinergic upregulation is already present in early PD with and without cognitive impairment, mainly distributed in the posterior cortical regions^33,34^. However, cholinergic activity is decreasing in PD patients with cognitive impairment as the disease progresses^35,36^. These findings are in line with our result that the aberration of the surface area of the OLF in PD patients is not related to motor dysfunction but is related to cognitive dysfunction. The above research indicated that the cerebrum of early PD patients maintains cognitive functioning through the mechanism of “compensation”, including the “compensated hyperplasia” and the upregulation of cholinergic activity.

This study had some limitations. Firstly, only single-modal MRI analysis using T1 structural images was performed. However, by T1WI, neuroimaging studies provide important insights into the anatomy and pathology of cerebral disease, which is also common in the diagnostic, differential, and predictive research of PD^37,38^. It should further contain functional MRI, such as resting state or dynamic functional MRI and diffuse tensor imaging, to explore the alteration of function and microstructure of WM and the possibility to predict FOG. Secondly, one of the most significant limitations, aside from sample size, is the limited amount of clinical data. A more comprehensive clinical assessment and maybe a more sensitive cognitive scale would better support the correlation analysis between structural features and clinical scales. However, the clinical data provided by PPMI is limited. In the future, we could try to build our own database to enlarge the clinical scales. Thirdly, universality is an issue. Although we applied diverse machine-learning algorithms, the results might differ if different machine-learning methods are applied with different cohorts, which needs to be considered when interpreting this study. Fourth, one of the inclusion criteria was that PD patients include both T1WI and diffusion-weighted images (DTI) data at baseline. However, only T1WI was used to calculate the brain structure indicators in this study. We will include the DTI data of the same group of subjects in further research to perform white matter microstructure attributes, including fractional anisotropy, mean diffusion, and even brain network attributes. What’s more, the participants in the PPMI database were mainly from European and American populations. Therefore, our results need to be verified in larger Asian populations.

In conclusion, we found increasing the cortical surface area in the olfactory cortex in early drug-naïve PD patients, suggesting that the OLF exhibits predominantly cortical expansion in early PD and is associated with abnormal cognitive function. T1WI morphometric markers, including parts of the occipital and frontal lobes and the limbic system, have the potential to help predict future FOG in patients with early PD at an individual level, which has higher predictive performance combined with clinical investigations.

## Methods

### Study design and participants

All data used in the current study were downloaded in May 2020 from the Parkinson’s Progressive Marker’s Initiative (PPMI) database (www.ppmi-info.org/), a longitudinal, observational, multicenter study combining advanced imaging, clinical and biologic data to identify biomarkers of PD progression^39^. Only subjects with clinical and laboratory measures, T1WI and DTI obtained on 3.0 T MRI scanners at baseline, were enrolled in our study. All participants were recruited between 2011 and 2015 from 12 sites (Supplementary Table 1).

According to the PPMI inclusion criteria (www.ppmi-info.org/study-design/research-documents-and-sops/), all participants with PD should meet the following criteria: (1) at least 30 years old when first diagnosed with PD, (2) a diagnosis of PD for at least two years on the screening date, (3) a significant dopamine transporter deficit confirmed by dopamine uptake transporter (DAT) scan, (4) Hoehn and Yahr Scale (H&Y) stage I or II at baseline, and (5) be untreated for PD at baseline. HCs enrolled in the study met the criteria, as they were at least 30 years old at the enrollment date, had no history of any observable neurologic deficits, had first-degree family members with PD, and had a score on the Montreal Cognitive Assessment (MOCA) of ≥26. The criteria above yielded 171 participants with drug-naïve PD and 77 HCs who were used for further analysis and quality control.

### Ethical approval

The PPMI study is registered at ClinicalTrials.gov (NCT01141023). This study was approved by the ethics committees: the Institutional Review Board of all participating sites for PPMI. Written informed consent was obtained from all individuals participating in the study.

### Clinical and laboratory assessments

To fully comprehend the possible mechanism for the development of PD into FOG, we included clinical and laboratory indicators as follows (Table 1):

(1) thirteen clinical assessments (all PD patients were drug-naïve): Rapid eye movement sleep behavior disorder screening questionnaire (RBDSQ), University of Pennsylvania Smell Identification Test (UPSIT), State-trait Anxiety Inventory (STAI), Geriatric Depression Scale (GDS), Questionnaire for Impulsive-Compulsive Disorders in Parkinson’s Disease (QUIP), Unified Parkinson’s Disease Rating Scale Part 1–3 (UPDRS I–III), Tremor score, PIGD score and Scales for Outcomes in Parkinson’s Disease—Autonomic (SCOPA-AUT). All clinical evaluations above were performed for every participant by the site investigators.

(2) nine laboratory assessments: Both urates of the blood sample and α-synuclein (α-syn), Aβ 1–42, total tau (t-tau), and p-tau concentrations of the CSF sample were analyzed in this study. Sample acquisition and measurement methods are available on the PPMI website (www.ppmi-info.org/study-design/research-documents-and-sops/).

### Assessment of FOG

The presence of FOG was defined if the score was ≥1 on UPDRS item 2.13 or item 3.11 anytime during the follow-up period in the participants with PD in a random motor state^11,17^. The time to occurrence of FOG was calculated as the number of months since study enrollment. Eight participants with PD already having a score ≥1 at baseline were excluded from this research.

### Imaging acquisition

Based on the PPMI database imaging protocols, whole-brain structural T1WI of these participants performed at various sites on a 3.0 T Siemens (TIM Trio and Verio) scanner (Erkangen, Germany) was used for further analysis in this study. For each participant, MPRAGE T1W images were acquired with the following parameters: repetition time (TR) = 2300 ms, echo time (TE) = 2.98 ms, field of view (FOV) = 240 mm × 256 mm, flip angle (FA) = 9°, and voxel size = 1 × 1 × 1 mm^3^. The details of the data acquisition parameters are available on the PPMI website (http://www.ppmi-info.org/study-design/ research-documents-and-sops/).

### Construction of structural morphological features

Following a visual inspection, nine scans (four HCs and five PD patients) were removed due to cerebral insufficiency and/or blurring and/or motion artifacts. All structural morphological features were generated through the CIVET pipeline (version 2.1), which was developed at the Montreal Neurological Institute (MNI). MRI images were automatically segmented into bilateral regions of interest, with cortical thickness, surface area, surface mean curvature, and GM volumes calculated at each region according to the Anatomical Automatic Labeling (AAL)_90_1-mm atlas^40^, with WM volumes calculated at each region according to the WM John Hopkins University Atlas JHU-ICBM-tracts-maxprob-thr25-1 mm^41^. The details of the pipeline processing steps are described in the supplementary methods.

### Predictive models of freezing of gait

#### Elastic net-support vector machine model and weighting factors

According to the AAL atlas and the WM JHU atlas, regional averaged cortical thickness, surface area, surface mean curvature, GM volumes, and WM volumes were formatted as structural morphological features of a length of 332 for each subject. The ROI-wise features were used instead of voxel-wise features because the former strategy was more adaptable to different imaging parameters and could significantly reduce the feature dimension. Selecting the most predictive features was essential to obtain a concise classification model and avoid overfitting. It is known that the elastic net often outperforms the lasso while enjoying a similar sparsity of representation. In addition, the elastic net not only encourages a grouping effect, where highly correlated predictors tend to be in or out of the model together but are also especially applicable when the number of predictors is much larger than the number of observations^42^. A sparse feature learning method based on an elastic net with different parameters was used for feature selection in this study. The elastic net estimator model is defined as follows:

$$\hat \beta = {\rm{arg}}\mathop {{{\rm {min}}}}\limits_\beta \left\| {Y - \omega ^{\rm {T}}X} \right\| + \lambda _1\left\| \omega \right\|_1 + \lambda _2\left\| \omega \right\|_2$$

where *Y* is the group label, *Y* = 1 or 2, *X* is the feature, *λ*_*ⅈ*_ is the regularization parameter and *ω* is the coefficient of each parameter.

We predicted FOG with features selected from the elastic net estimator model using linear support vector machine (EN-SVM) classifiers with a nested 10-fold cross-validation strategy. To explore whether different proportions of future FOG to non-FOG occurrence affect model performance, we put different proportions of future FOG and non-FOG patients into training and test sets, including 4:6 (original proportion), 5:5, and 3:7. Then, structural morphological feature-based FOG classification was carried out by a new SVM classifier trained with the optimal feature set and evaluated by outer 10-fold cross-validation. The accuracy (ACC), sensitivity (SEN), specificity (SPE), and area under the receiver operating characteristic (ROC) curve (AUC) were obtained to evaluate the classification performance. In addition, clinical and laboratory assessments were then added to evaluate their contribution to the prediction model of FOG.

To determine the features that make the greatest contribution to the FOG prediction model, a transformation of the testing sample to the features when generating the prediction sample was defined as the weighting factor. That is, the complex feature points in the high-dimensional space were projected onto the low-dimensional plane through the weight transformation. As a result, the smaller the absolute value of the weight corresponding to these features was, the less important the feature was. That is, the higher the absolute value of the feature corresponding weight was, the more obvious the enhancement effect was, and the more important the contribution to the classification was.

#### Feature selection and training of machine learning models

Moreover, we compared the prediction performance of different machine-learning methods using the GFS with matFR toolbox^43^, and four machine-learning models: LSVM, K near neighbor (MNN), naïve Bayes (NB), and linear discriminant analysis (LDA). The same as above, the potential features, including 13 clinical variables, nine CSF indicators, and 332 regional morphological images, were normalized by *z*-score. We randomly divided the dataset into a training set and a test set with a ratio of 5:5. In the training set, 53 cases included both the PD patients used to develop the FOG sample and the PD patients who were not used to develop the FOG sample. Features selection based on GFS was used to train the machine learning models. All procedures were repeated 50 times at random.

### Statistical analysis

All categorical variables and continuous variables included in this study were compared using Pearson chi-square tests and Mann–Whitney *U* tests, respectively. Statistical analysis was performed using SPSS 25.0 (IBM Corp., Armonk, NY), and a value of *P* < 0.05 was regarded as statistically significant.

Two-sample *t*-tests were used to compare the structural morphology measurements between the PD patients and HCs, as well as future FOG and non-FOG. To correct for multiple comparisons when using neuroimaging data, the false discovery rate (FDR) was used, with a threshold of *P* < 0.05. Age, sex, and site were used as covariates. Spearman correlation analyses were adopted to detect relationships between structural morphological features with statistically significant differences and clinical and laboratory assessments, with a *P*-value Bonferroni correction for multiple comparisons.

### Reporting summary

Further information on research design is available in the Nature Research Reporting Summary linked to this article.

## Supplementary information

Supplementary materials

Reporting Summary

## Data Availability

All data reported in this article are available in the PPMI database (http://ppmi-info.org). All codes used in this article are available upon reasonable request from the corresponding author.
