# Supplementary material for: Baseline cerebral structural morphology predict freezing of gait in early drug-naïve Parkinson’s disease
Source: NPJ Parkinsons Dis. 2022 Dec 29;8:176. doi: 10.1038/s41531-022-00442-4 (PMC9800563; doi:10.1038/s41531-022-00442-4)
Supplement: Supplementary file 1 — Supplementary materials [file 41531_2022_442_MOESM1_ESM.pdf]

Supplementary Material

Supplementary methods: Construction process of structural morphological features

The main pipeline processing steps were described below: i) The native three-dimensional T_1 images of each subject were corrected for non-uniformity artifacts using the N3 algorithm; ii) Classification of the grey matter (GM), white matter (WM) and CSF was performed using the INSECT algorithm; iii) The Constrained Laplacian-based Anatomic Segmentation with Proximity (CLASP) algorithm was applied to generate a model of the cortical surface, including 40,962 vertices and 81,920 triangular meshes per hemisphere; iv) Hemispheric surfaces were generated for both the WM/GM interface and GM/CSF interface; v) surfaces for each hemisphere were non-linearly registered to an average surface created from the ICBM152 brain template; vi) A reverse linear transformation was carried out on each subject's images, and cortical thickness estimations were calculated at each cortical point in native space using the tlink metric; vii) Subjects' surface maps, including cortical thickness, surface area, GM surface mean curvature, were blurred using a 20-millimeter full width at half maximum surface-based diffusion smoothing kernel; viii) Process voxel-based morphometry (VBM) files to calculate the GM volumes and WM volumes; ix) Blurring kernel size in 8 mm for volume; x) Cortical thickness, surface area, surface mean curvature and GM volumes were calculated at each region according to the Anatomical Automatic Labeling (AAL)_90_1-mm atlas, while WM volumes were calculated at each region according to the WM John Hopkins University Atlas JHU-ICBM-tracts-maxprob-thr25-1 mm.

Supplementary Figure 1 Differences in surface area between Parkinson's disease (PD) and healthy controls (HCs) and its relationship with the Montreal Cognitive Assessment (MOCA). (a) The bilateral olfactory cortex (OLF) showed significant increasing surface area in PD patients than in HC. (b) Only surface area of the left OLF was correlated with the MOCA. L = left; R = right.

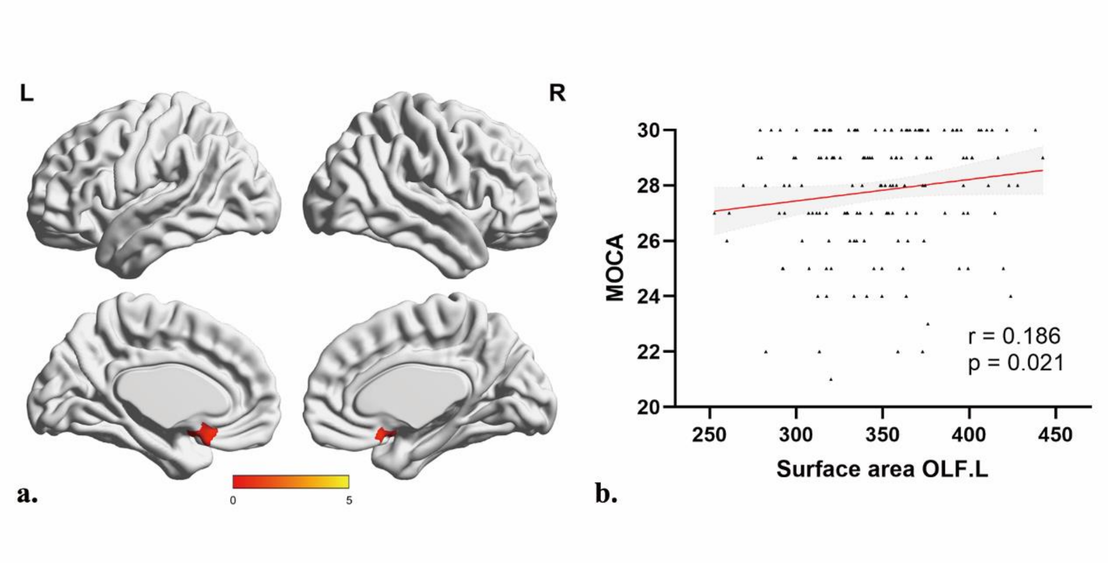

Supplementary Table 1: Participants numbers of each center.

Center number	HCs	Future NFOG	Future FOG	PD	Total
7	4	11	8	19	23
12	0	1	2	3	3
28	5	10	3	13	18
32	12	11	9	20	32
73	10	9	4	13	23
88	9	10	7	17	26
120	5	13	8	21	26
196	2	2	1	3	5
289	7	7	11	18	25
290	10	13	9	22	32
291	8	3	2	5	13
304	1	2	2	4	5
Total 12	73	92	66	158	231

HCs = healthy controls; PD = Parkinson's disease; FOG = freezing of gait; NFOG = non-freezing of gait.

Supplementary Table 2: EV-SVM model performance for different proportions of future NFOG and FOG in train and test sets.

Features	Future NFOG : FOG	AUC	ACC	SEN	SPE
BF	4:6	0.7	0.69	0.55	0.84
	5:5	0.67	0.66	0.59	0.78
	3:7	0.76	0.75	0.85	0.66
SF	4:6	0.73	0.72	0.7	0.74
	5:5	0.83	0.76	0.89	0.67
	3:7	0.86	0.79	0.77	0.81

All	4:6	0.77	0.78	0.74	0.83
	5:5	0.91	0.84	0.94	0.71
	3:7	0.91	0.84	0.90	0.83

FOG = freezing of gait; NFOG = non-freezing of gait; AUC = area under curve; ACC = accuracy; SEN = sensitivity; SPE = specificity; BF = both clinical and laboratory features; SF = structural features.

Supplementary Table 3: Regions with increased surface area in PD patients.

Group	Region	x	y	z	T-value	Peak voxel p	FDR corrected p	Size (voxel)
PD > HC	OLF.L	82	141	61	3.536	0.039	0.05	87
	OLF.R	100	142	61	3.317	0.042	0.05	81

PD = Parkinson' s disease; HC = healthy controls; FDR = false discovery rate; OLF = olfactory cortex; L= left; R = right.

Supplementary Table 4: Aberrant structural brain regions in patients with future FOG compared with future NFOG at baseline.

Group	Region	x	y	z	T-value	Peak voxel p	Size (vertex/voxel)
	Cortical thickness						
NFOG > FOG	PCG.L	85	83	97	1.595	0.025	137
	Cortical mean curve						
NFOG < FOG	ROL.L	43	118	86	-1.642	0.013	302
	INS.L	55	133	75	-1.949	0.002	566
	CUN.R	104	47	100	-1.231	0.012	434
	MOG.R	127	46	91	-0.241	0.009	595
NFOG > FOG	SMA.R	99	126	134	1.323	0.028	666
	Surface area						
NFOG < FOG	SMA.R	99	126	134	-1.389	0.030	666
	SOG.R	114	45	103	-1.027	0.013	428
	PoCG.R	131	101	125	-0.505	0.032	1138
	SPG.L	67	66	131	-0.032	0.027	631
	SPG.R	116	67	134	-0.128	0.014	647
NFOG > FOG	MOG.R	127	46	91	0.171	0.024	595
	PoCG.L	48	103	121	0.050	0.031	1159
	PCUN.L	83	70	120	0.751	0.043	1079
	MTG.L	34	92	70	0.448	0.024	1439
	MTG.R	147	89	71	1.209	0.008	1356

	Grey matter volume						
NFOG < FOG	SMA.R	99	126	134	-0.943	0.039	666
	CUN.R	104	47	100	-0.339	0.015	434
	LING.R	106	59	68	-0.276	0.030	678
	IOG.L	54	48	64	-2.696	0.013	268
	IOG.R	128	44	64	-2.956	0.006	314
	FFG.L	59	86	52	-1.989	0.042	687

* None of the above structural measurements could be corrected by false discovery rate (FDR). FOG = freezing of gait; NFOG = non-freezing of gait; L = left; R = right; PCG = posterior cingulate gyrus; ROL = rolandic operculum; SMA = supplementary motor area; INS = insula; CUN = cuneus; MOG =middle occipital gyrus; SOG = superior occipital gyrus; PoCG = postcentral gyrus; SPG = superior parietal gyrus; PCUN = precuneus; MTG = middle temporal gyrus; LING = lingual gyrus; IOG = inferior occipital gyrus; FFG = fusiform gyrus.
