# Supplementary material for: Baseline cerebral structural morphology predict freezing of gait in early drug-naïve Parkinson’s disease
Source: NPJ Parkinsons Dis. 2022 Dec 29;8:176. doi: 10.1038/s41531-022-00442-4 (PMC9800563; doi:10.1038/s41531-022-00442-4)
Supplement: Supplementary file 2 — Reporting Summary [file 41531_2022_442_MOESM2_ESM.pdf]

nature portfolio

Corresponding author(s): Xinhua Wei

Last updated by author(s): Dec 8, 2022

Reporting Summary

Nature Portfolio wishes to improve the reproducibility of the work that we publish. This form provides structure for consistency and transparency in reporting. For further information on Nature Portfolio policies, see our Editorial Policies and the Editorial Policy Checklist.

Statistics

For all statistical analyses, confirm that the following items are present in the figure legend, table legend, main text, or Methods section.

n/a	Confirmed	
☐	☒	The exact sample size (n) for each experimental group/condition, given as a discrete number and unit of measurement
☐	☒	A statement on whether measurements were taken from distinct samples or whether the same sample was measured repeatedly
☐	☒	The statistical test(s) used AND whether they are one- or two-sided<br>*Only common tests should be described solely by name; describe more complex techniques in the Methods section.*
☐	☒	A description of all covariates tested
☐	☒	A description of any assumptions or corrections, such as tests of normality and adjustment for multiple comparisons
☐	☒	A full description of the statistical parameters including central tendency (e.g. means) or other basic estimates (e.g. regression coefficient) AND variation (e.g. standard deviation) or associated estimates of uncertainty (e.g. confidence intervals)
☐	☒	For null hypothesis testing, the test statistic (e.g. F, t, r) with confidence intervals, effect sizes, degrees of freedom and P value noted<br>*Give P values as exact values whenever suitable.*
☐	☒	For Bayesian analysis, information on the choice of priors and Markov chain Monte Carlo settings
☐	☒	For hierarchical and complex designs, identification of the appropriate level for tests and full reporting of outcomes
☐	☒	Estimates of effect sizes (e.g. Cohen's d, Pearson's r), indicating how they were calculated

Our web collection on statistics for biologists contains articles on many of the points above.

Software and code

Policy information about availability of computer code

Data collection	All data collected were downloaded in the Parkinson's Progressive Marker's Initiative (PPMI) database (www.ppmi-info.org/).
Data analysis	All structural morphological features were generated through the CIVET pipeline (version 2.1). Statistical analysis was performed using SPSS 25.0 (IBM Corp, Armonk, NY)

For manuscripts utilizing custom algorithms or software that are central to the research but not yet described in published literature, software must be made available to editors and reviewers. We strongly encourage code deposition in a community repository (e.g. GitHub). See the Nature Portfolio guidelines for submitting code & software for further information.

Data

Policy information about availability of data

All manuscripts must include a data availability statement. This statement should provide the following information, where applicable:

- Accession codes, unique identifiers, or web links for publicly available datasets
- A description of any restrictions on data availability
- For clinical datasets or third party data, please ensure that the statement adheres to our policy

All data reported in this article are available in the PPMI database (http://ppmi-info.org).

Human research participants

Policy information about studies involving human research participants and Sex and Gender in Research.

Reporting on sex and gender	We have reported the sex ratio in the Table 1.
Population characteristics	Age, sex and site were used as covariates.
Recruitment	Only subjects with clinical and laboratory measures, T1-weighted images (T1WI) obtained on 3.0 T MRI scanners at baseline, were enrolled in our study.
Ethics oversight	The PPMI study is registered at ClinicalTrials.gov (NCT01141023). This study was approved by the ethics committees: the Institutional Review Board of all participating sites for PPMI.

Note that full information on the approval of the study protocol must also be provided in the manuscript.

Field-specific reporting

Please select the one below that is the best fit for your research. If you are not sure, read the appropriate sections before making your selection.

☒ Life sciences ☐ Behavioural & social sciences ☐ Ecological, evolutionary & environmental sciences

For a reference copy of the document with all sections, see nature.com/documents/nr-reporting-summary-flat.pdf

Life sciences study design

All studies must disclose on these points even when the disclosure is negative.

Sample size	The criteria yielded 171 participants with drug-naïve PD and 77 HCs who were used for further analysis and quality control.
Data exclusions	According to the PPMI inclusion criteria (www.ppmi-info.org/study-design/research-documents-and-sops/), all participants with PD should meet the following criteria: (1) at least 30 years old when first diagnosed with PD, (2) a diagnosis of PD for at least two years on the screening date, (3) a significant dopamine transporter deficit confirmed by dopamine uptake transporter (DAT) scan, (4) Hoehn and Yahr Scale (H&Y) stage ? or ? at baseline, and (5) be untreated for PD at baseline. HCs enrolled in the study met the criteria, as they were at least 30 years old at the enrollment date, had no history of any observable neurologic deficits, had first-degree family members with PD, and had a score on the Montreal Cognitive Assessment (MOCA) of ≥ 26.
Replication	We use different algorithms to develop prediction models, each of which is replicated or cross-validated.
Randomization	We randomly the samples into the training set and test set in different proportions.
Blinding	All investigators were blinded to group allocation during data analysis.

Reporting for specific materials, systems and methods

We require information from authors about some types of materials, experimental systems and methods used in many studies. Here, indicate whether each material, system or method listed is relevant to your study. If you are not sure if a list item applies to your research, read the appropriate section before selecting a response.

Materials & experimental systems

n/a	Involved in the study	
☒	☐	Antibodies
☒	☐	Eukaryotic cell lines
☒	☐	Palaeontology and archaeology
☒	☐	Animals and other organisms
☐	☒	Clinical data
☒	☐	Dual use research of concern

Methods

n/a	Involved in the study	
☒	☐	ChIP-seq
☒	☐	Flow cytometry
☐	☒	MRI-based neuroimaging

Clinical data

Policy information about clinical studies

All manuscripts should comply with the ICMJE guidelines for publication of clinical research and a completed CONSORT checklist must be included with all submissions.

Clinical trial registration	The PPMI study is registered at ClinicalTrials.gov (NCT01141023).
Study protocol	All data used in the current study were downloaded in May 2020 from the Parkinson's Progressive Marker's Initiative (PPMI) database (www.ppmi-info.org/).
Data collection	All data used in the current study were downloaded in May 2020 from the Parkinson's Progressive Marker's Initiative (PPMI) database (www.ppmi-info.org/)
Outcomes	Here, we developed a model that could predict the occurrence of FOG at the individual level using machine learning with clinical assessments, laboratory tests and cerebral structural imaging information of early drug-naïve PD patients. As a secondary objective, we explored the morphological alterations of the cerebrum in early drug-naïve PD patients and their relationship with clinical and laboratory assessments.

Magnetic resonance imaging

Experimental design

Design type	structual imaging
Design specifications	structual imaging
Behavioral performance measures	structual imaging

Acquisition

Imaging type(s)	structual
Field strength	3.0 T
Sequence & imaging parameters	MPRAGE T1W images were acquired with the following parameters: repetition time (TR) = 2300 ms, echo time (TE) = 2.98 ms, field of view (FOV) = 240 mm × 256 mm, flip angle (FA) = 9°, and voxel size = 1 × 1 × 1 mm3.The details of the data acquisition parameters are available on the PPMI website (http://www.ppmi-info.org/study-design/ research-documents-and-sops/).
Area of acquisition	MRI images were automatically segmented into bilateral regions of interest, with cortical thickness, surface area, surface mean curvature and GM volumes calculated at each region according to the Anatomical Automatic Labeling (AAL)_90_1-mm atlas, with WM volumes calculated at each region according to the WM John Hopkins University Atlas JHU-ICBM-tracts-maxprob-thr25-1 mm.

Diffusion MRI ☐ Used ☒ Not used

Preprocessing

Preprocessing software	All structural morphological features were generated through the CIVET pipeline (version 2.1).
Normalization	The main pipeline processing steps were described below: ?) The native three-dimensional T1 images of each subject were corrected for non-uniformity artifacts using the N3 algorithm; ?) Classification of the grey matter (GM), white matter (WM) and CSF was performed using the INSECT algorithm; ?) The Constrained Laplacian-based Anatomic Segmentation with Proximity (CLASP) algorithm was applied to generate a model of the cortical surface, including 40,962 vertices and 81,920 triangular meshes per hemisphere; ?) Hemispheric surfaces were generated for both the WM/GM interface and GM/CSF interface; ?) surfaces for each hemisphere were non-linearly registered to an average surface created from the ICBM152 brain template; ?) A reverse linear transformation was carried out on each subject's images, and cortical thickness estimations were calculated at each cortical point in native space using the tlink metric; ?) Subjects' surface maps, including cortical thickness, surface area, GM surface mean curvature, were blurred using a 20-millimeter full width at half maximum surface-based diffusion smoothing kernel; ?) Process voxel-based morphometry (VBM) files to calculate the GM volumes and WM volumes; ?) Blurring kernel size in 8 mm for volume; x) Cortical thickness, surface area, surface mean curvature and GM volumes were calculated at each region according to the Anatomical Automatic Labeling (AAL)_90_1-mm atlas, while WM volumes were calculated at each region according to the WM John Hopkins University Atlas JHU-ICBM-tracts-maxprob-thr25-1 mm.
Normalization template	Cortical thickness, surface area, surface mean curvature and GM volumes were calculated at each region according to the Anatomical Automatic Labeling (AAL)_90_1-mm atlas, while WM volumes were calculated at each region according to the WM John Hopkins University Atlas JHU-ICBM-tracts-maxprob-thr25-1 mm.
Noise and artifact removal	Following a visual inspection, nine scans (four HCs and five PD patients) were removed due to cerebral insufficiency and/or blurring and/or motion artifacts.

Volume censoring

Following a visual inspection, nine scans (four HCs and five PD patients) were removed due to cerebral insufficiency and/or blurring and/or motion artifacts.

Statistical modeling & inference

Model type and settings

The elastic net estimator model is defined as follows:
β ̂=arg?(min) ┬β ||(Y-ω^T X||+λ_1 ||ω||_1+λ_2 ||ω)||_2 ?
where Y is the group label, Y = 1 or 2, X is the feature, λ_? is the regularization parameter and ω is the coefficient of each parameter.
We predicted FOG with features selected from the elastic net estimator model using linear support vector machine (EN-SVM) classifiers with a nested 10-fold cross-validation strategy. Moreover, we compared the prediction performance of different machine learning methods using the GFS with matFR toolbox39, and four machine learning models: LSVM, K near neighbor (MNN), naïve Bayes (NB) and linear discriminant analysis (LDA).

Effect(s) tested

Two-sample t tests were used to compare the structural morphology measurements between the PD patients and HCs, as well as future FOG and non-FOG. To correct for multiple comparisons when using neuroimaging data, the false discovery rate (FDR) was used, with a threshold of $P < 0.05$. Age, sex and site were used as covariates. Spearman correlation analyses were adopted to detect relationships between structural morphological features with statistically significant differences and clinical and laboratory assessments, with a p-value Bonferroni correction for multiple comparisons.

Specify type of analysis: ☐ Whole brain ☒ ROI-based ☐ Both

Anatomical location(s)

Cortical thickness, surface area, surface mean curvature and GM volumes were calculated at each region according to the Anatomical Automatic Labeling (AAL)_90_1-mm atlas, while WM volumes were calculated at each region according to the WM John Hopkins University Atlas JHU-ICBM-tracts-maxprob-thr25-1 mm.

Statistic type for inference
(See Eklund et al. 2016)

Process voxel-based morphometry (VBM) files to calculate the GM volumes and WM volumes; Blurring kernel size in 8 mm for volume; Cortical thickness, surface area, surface mean curvature and GM volumes were calculated at each region according to the Anatomical Automatic Labeling (AAL)_90_1-mm atlas, while WM volumes were calculated at each region according to the WM John Hopkins University Atlas JHU-ICBM-tracts-maxprob-thr25-1 mm.

Correction

FDR

Models & analysis

n/a	Involved in the study
☒	☐ Functional and/or effective connectivity
☒	☐ Graph analysis
☐	☒ Multivariate modeling or predictive analysis

Multivariate modeling and predictive analysis

Independent variables includd 13 clinical variables, nine CSF indicators and 332 regional morphological images. We predicted FOG with features selected from the elastic net estimator model using linear support vector machine (EN-SVM) classifiers with a nested 10-fold cross-validation strategy. Moreover, we compared the prediction performance of different machine learning methods using the GFS with matFR toolbox39, and four machine learning models: LSVM, K near neighbor (MNN), naïve Bayes (NB) and linear discriminant analysis (LDA).
